# Can Pyrolysis Oil Be Used as a Feedstock to Close the Gap in the Circular Economy of Polyolefins?

**DOI:** 10.3390/polym15040859

**Published:** 2023-02-09

**Authors:** Berrak Erkmen, Adem Ozdogan, Ayhan Ezdesir, Gokhan Celik

**Affiliations:** 1 SOCAR Turkey R&D and Innovation Inc., SOCAR Turkey, Izmir 35800, Turkey; 2 Refinery and Petrochemicals Business Unit, SOCAR Turkey, Izmir 35800, Turkey; 3 Chemical Engineering Department, Middle East Technical University, Ankara 06800, Turkey

**Keywords:** pyrolysis, pyrolysis oil, naphtha, waste plastic, chemical recycling, polyolefins, bromine number, PIONA, circular economy

## Abstract

Plastics are engineering marvels that have found widespread use in all aspects of modern life. However, poor waste management practices and inefficient recycling technologies, along with their extremely high durability, have caused one of the major environmental problems facing humankind: waste plastic pollution. The upcycling of waste plastics to chemical feedstock to produce virgin plastics has emerged as a viable option to mitigate the adverse effects of plastic pollution and close the gap in the circular economy of plastics. Pyrolysis is considered a chemical recycling technology to upcycle waste plastics. Yet, whether pyrolysis as a stand-alone technology can achieve true circularity or not requires further investigation. In this study, we analyzed and critically evaluated whether oil obtained from the non-catalytic pyrolysis of virgin polypropylene (PP) can be used as a feedstock for naphtha crackers to produce olefins, and subsequently polyolefins, without undermining the circular economy and resource efficiency. Two different pyrolysis oils were obtained from a pyrolysis plant and compared with light and heavy naphtha by a combination of physical and chromatographic methods, in accordance with established standards. The results demonstrate that pyrolysis oil consists of mostly cyclic olefins with a bromine number of 85 to 304, whereas light naphtha consists of mostly paraffinic hydrocarbons with a very low olefinic content and a bromine number around 1. Owing to the compositional differences, pyrolysis oil studied herein is completely different than naphtha in terms of hydrocarbon composition and cannot be used as a feedstock for commercial naphtha crackers to produce olefins. The findings are of particular importance to evaluating different chemical recycling opportunities with respect to true circularity and may serve as a benchmark to determine whether liquids obtained from different polyolefin recycling technologies are compatible with existing industrial steam crackers’ feedstock.

## 1. Introduction

Plastics are ubiquitous and versatile materials, and they are used in all aspects of modern civilization at tremendous quantities. Four hundred million tons (400 Mt) of plastics are produced each year. It is estimated that the production of plastics will only increase and exceed one million tons by the end of 2050 [1,2,3,4,5]. After being used for their intended purposes, plastics complete their useful lifecycles and are discarded. Some plastics such as straws and utensils are produced for single use with a useful lifetime between seconds to minutes, whereas some plastics such as shampoo bottles or garbage bins can be used for longer durations with a useful lifetime between weeks to years. Regardless of the time scale at which plastics are discarded, their waste management becomes crucial because they degrade slowly. For instance, it takes up to 200 years for a plastic straw to degrade naturally [6].

Although a simple comparison between useful lifetimes and natural degradation durations points out the importance of waste management of plastics to prevent their haphazard accumulations in the environment, existing infrastructures cannot cope with the waste plastics, causing one of the biggest environmental challenges facing humankind: plastic pollution [1,2,3,5]. Global mass production analysis [7] of plastics shows that out of eight billion metric tons of plastics ever produced by 2017, 70% of them ended up in landfills or in aquatic life polluting our planet. Another 14% of them were incinerated to produce energy. However, this option causes the emission of greenhouses gases such as CO_2_ at large volumes. The remaining 16% was recycled to obtain lower-value materials with a low efficacy [8]. These methods are not environmentally friendly and cause continuous consumption of natural resources (from crude oil or natural gas to plastics to waste plastics), favoring a linear economy.

Plastics are, however, engineering marvels with high energy and chemical content. Producing 400 Mt of plastics approximately requires a consumption of 7% of crude oil and natural gas produced [3]. Considering the expensive and scarce fossil fuel resources used in plastic production, it is unfortunate to waste these resources for creating waste, polluting the environment, or losing their value to low-quality recycled products, which oftentimes cannot be recycled after several life cycles [9,10,11]. This process can be considered as an example of a linear economy because resources are linearly converted to plastics and eventually to waste. Although the linear system creates a massive economic value by producing and selling plastics, the result is the global waste plastic problem and the loss of value and resources. New and innovative approaches are needed to replace the linear economy of plastics by the circular economy. There are currently many efforts to break the linear system and repurpose or upcycle waste plastic to value-added products. One of the most recent examples is the chemical conversion of single-use PE to lubricants by a catalytic upcycling process using platinum nanoparticles supported on perovskites [8,12]. Producing lubricants from PE is of particular importance because they can be successfully recycled with infinite turns, creating a circular carbon economy [13,14]. The study also demonstrates that lubricants perform as well as their commercial counterparts and the conversion of PE to lubricants is economically feasible.

Another viable option that can truly close the gap in the circular economy of polyolefins is the chemical conversion of waste to feedstock that are used to produce virgin polyolefins. Polyolefin precursors are produced from steam cracking of naphtha [15]. Naphtha is a hydrocarbon fraction, which usually constitutes 15–30% by weight of crude oil and has a boiling point range between 30 °C and 200 °C. It contains hydrocarbon molecules with 5–12 carbon atoms, mostly including saturated hydrocarbons such as paraffins and naphthenes with minor compounds including olefins and aromatics [16]. There are two types of naphtha blends produced from the distillation of crude oil in the refineries: (i) heavy naphtha which consists of mainly alkanes and cycloalkanes with a boiling point of 70 to 200 °C and is used to produce aromatics [17], and (ii) light naphtha (also known as low-boiling naphtha) which consists of mostly pentane and hexane derivatives and is fed to the steam cracker unit to produce polyolefin precursors [18].

If the objective is to obtain virgin polyolefins from the waste to achieve circularity, waste polyolefins should be converted to a compound that resembles naphtha and fed to the cracker unit. This is only possible by chemical recycling, a process that breaks down longer polymeric chains into smaller units which can be recycled into a range of useful materials. Various chemical recycling methods, such as pyrolysis, gasification, and hydrothermal processing, can be used to convert plastic wastes into gases, fuels, and other compounds. Yet, pyrolysis is a more viable choice if the intended product is a liquid that can be fed to the steam crackers [19,20].

Pyrolysis is the thermal degradation of hydrocarbon-based feedstock materials by heating in an oxygen-free environment at high temperatures (300–700 °C). Because of heating, large-chain polymers decompose into smaller hydrocarbons. The pressure is typically atmospheric although it can also be performed under vacuum. The pyrolysis performance also depends on the properties of the feedstock such as molecular structure including chain irregularities, branching, initiators, chain lengths, and crystallinity, etc. When a pyrolysis-like technology is applied, the carbon number of the cleaved polymers decrease, eventually reaching a point where it exists as liquid in pseudo-equilibrium with its vapor in the pyrolysis reactor. The properties of the liquid obtained by pyrolysis can be very similar to conventional fuels (in terms of energy content, octane and cetane number, and other physical properties such as density, viscosity, flash-point, etc.). What in turn determines the properties of these liquids is the chemical composition of the liquid (including aromatic content, distillation range, paraffinic content, etc.) [21,22,23,24,25].

Herein, the suitability of pyrolysis as a stand-alone chemical recycling technique for producing the precursors for virgin polyolefins is examined. Liquid products obtained from the noncatalytic pyrolysis of polypropylene at two different temperatures were analyzed to obtain physical properties and chemical composition by several chromatography techniques, in comparison to two different naphtha mixtures, namely light naphtha and heavy naphtha. Liquid samples were then distilled under vacuum and fractionated to examine if a portion of the pyrolysis oil can be used as naphtha. The results demonstrate that, although there is a very small fraction of pyrolysis oil consisting of saturated alkanes and naphthenes, pyrolysis oil obtained from PP exhibits distinct compositional differences than naphtha and cannot be used as a substitute for it.

## 2. Materials and Methods

Pyrolysis oils were produced by the thermal non-catalytic degradation pyrolysis of polypropylene in two different temperature ranges. The polypropylene used in this study are bigbag sacks. ‘Py oil-1’ represents the liquid product from the reactor at 270–300 °C, and ‘Py oil-2’ represents the liquid product taken from the reactor at 370–400 °C. The properties of light and heavy naphtha samples were taken from the analysis results of the naphtha feedstocks that used as feedstock inputs in the Ethylene and Aromatics plants of PETKİM, respectively.

Density measurements were performed according to the American Society for Testing and Materials (ASTM) D4052 22-Standard Test Method for Density, Relative Density, and the American Petroleum Institute (API) Gravity of Liquids by Digital Density Meter. Vacuum distillation results were determined with the ASTM D1160 18-Standard Test Method for Distillation of Petroleum Products at Reduced Pressure. Total sulfur amount of the samples was measured with the ASTM D5453 19a-Standard Test Method for Determination of Total Sulfur in Light Hydrocarbons, Spark Ignition Engine Fuel, Diesel Engine Fuel, and Engine Oil by Ultraviolet Fluorescence.

To understand the boiling point distribution and the amount of the naphtha fraction of the pyrolysis oils, simulated distillation (SIMDIST) analysis ASTM D7169 was performed and boiling points were determined according to the ASTM D86-Standard Test Method for Distillation of Petroleum Products and Liquid Fuels at Atmospheric Pressure. SIMDIST is a gas chromatography (GC) method applied to characterize and separate petroleum fractions and products based on their boiling point. The principle of SIMDIST is based on a combination of traps for hydrocarbons with a similar functional group, separating columns and a hydrogenator for olefins. Atmospheric distillation is associated with the volumetric composition, energy content, and boiling range distribution of fuels and petroleum products.

Alkane profile of the samples were also obtained to examine carbon number distribution. Bromine number values of the samples were calculated by applying the method from the ASTM D1159-07-Standard Test Method for Bromine Numbers of Petroleum Distillates and Commercial Aliphatic Olefins by Electrometric Titration and compared with light and heavy naphtha results.

To examine the chemical compositions, PIONA (acronym for n-paraffins, iso-paraffins, olefins, naphthenes and aromatics) analysis was performed on pyrolysis oils fractionated up to 210 °C. This temperature was chosen to prevent column clogging in the PIONA analysis. PIONA is a unique method used in the refinery and petrochemical industries. It is a multi-dimensional chromatography technology containing seven separation columns. For each mode, all settings are preprogrammed to enable automatic column switching and temperature control. The analyzer system is specified according to the following standard methods: Euopean Norm International Organization for Standardization (EN ISO) 22854 and ASTM D6839.

## 3. Results and Discussion

**Figure 1:**
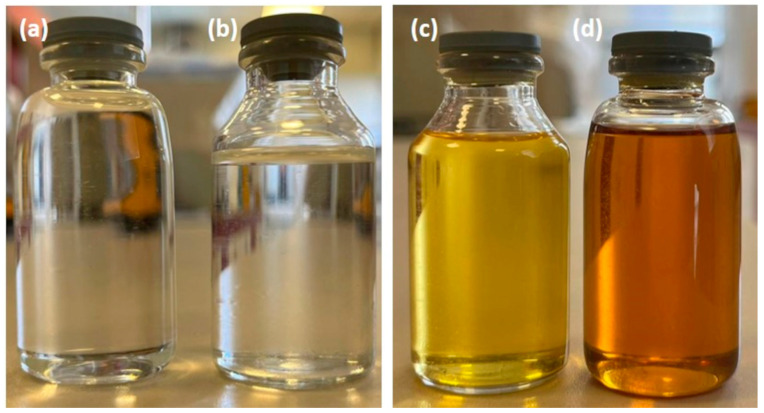
(**a**) Light naphtha, (**b**) heavy naphtha, (**c**) Py oil-1; the oil collected between 270–300 °C, (**d**) Py oil-2; the oil collected between 370–400 °C.

Two different pyrolysis oils, shown in Figure 1, were studied in comparison with light and heavy naphtha to examine the suitability of using pyrolysis oil for producing polyolefins to close the gap in the circularity of plastics. The first observed difference is the color of the samples. Py oil-1 is yellow, Py oil-2 is orange, and light and heavy naphtha are colorless.

All four liquids, two different pyrolysis oils and naphtha mixtures, have similar density values (Table 1) at 15 °C, as determined by the ASTM D4052 method. Pyrolysis oils have a density of 0.78-0.79 g/cm^3^, whereas the naphtha mixtures are slightly lighter having density in the range of 0.67 to 0.72 g/cm^3^ for light naphtha and a minimum density of 0.73 g/cm3 for heavy naphtha. In addition to density, total sulfur amount is determined by ASTM D5453. The total sulfur content was determined to be higher in the Py oil-1 sample collected at lower temperatures compared with the naphtha samples, and within the desired specification values in the Py oil-2 sample collected at higher temperatures. Yet, it should be noted that the purity of pyrolysis oils depends on the purity of the waste plastics. If the waste plastic is contaminated with sulfur-containing compounds, pyrolysis mixtures will contain higher amount of sulfur.

The boiling point fractionation was determined with vacuum distillation by the ASTM D1160 method and tabulated in Table 1. Vacuum distillation results of light naphtha and heavy naphtha are similar, albeit a slightly lower initial boiling point (IBP) for light naphtha. Vacuum distillation results of pyrolysis oils, however, show major differences than naphtha blends. IBPs of pyrolysis oils are slightly higher than naphtha mixtures. The required temperature for 90% fractionation of both pyrolysis oils is around 380–385 °C, significantly higher than that of naphtha mixtures, 90% of which fractionate at a minimum of 170 °C.

The fractionation of pyrolysis oils and naphtha liquids was also performed with simulated distillation (SIMDIST) to determine the volumetric fraction of pyrolysis oil that is similar to naphtha, based on where the final boiling point (FBP) of light and heavy naphtha are located in the distillation results of pyrolysis oils. This information is then used for determining the volumetric percentage of naphtha-like liquid in pyrolysis oils. The SIMDIST results in Table 2 show that light naphtha-like composition is 10–15% in Py oil-1 and 5–10% in Py-oil 2, whereas heavy naphtha-like composition is 30–40% in both Py oil-1 and Py oil-2.

**Figure 2:**
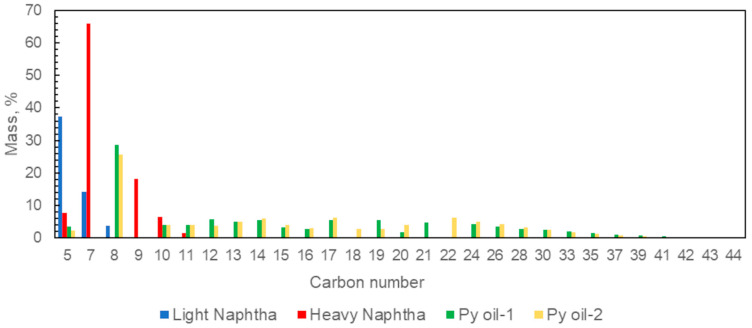
Alkane profile of the samples.

In Figure 2, the alkane profile of the samples based on the carbon numbers are illustrated. The C8 fraction had the highest value in the pyrolysis oil samples. These values were determined as 28.7% for Py oil-1 and 25.6% for Py oil-2. The carbon number distribution of the pyrolysis oils varied in the range of C5–C44.

The distillation studies and alkane distribution analysis point out that naphtha mixtures have a significantly lower number of carbons than pyrolysis oils. This is a major difference between naphtha and pyrolysis oil that may limit using the latter as a substitute for the former. Existing pyrolysis technologies are, however, equipped with a condenser that helps with decreasing the carbon number to the desired range. For instance, the patent disclosed by BlueAlp Innovations B.V. technology for chemical recycling of plastics describes the use of a partial condenser, which controls the composition of the pyrolyzed gas by the condenser temperature. In addition, pyrolysis oil can be distillated to obtain the desired fraction for downstream operations [26].

The carbon range of pyrolysis oil could be brought to the similar range with naphtha. What remains as a huge challenge is the compositional differences. To examine the compositional differences, the bromine number of each liquid was measured and given in Table 3. The number of grams of bromine that will react with 100 g of the specimen under the conditions of the test is defined as the bromine number. The bromine number quantifies and indicates the aliphatic unsaturated fraction of the petroleum products. By using this method, an estimation of the percentage of olefins in petroleum distillates boiling up to approximately 315 °C can be obtained, albeit at a lower precision above the bromine number of 185 [27]. Py oil-2 has a higher bromine number (304) than Py oil-1 (85), and hence, higher olefinic content. Mangest et al. reported that straight-chain olefins, branched-chain olefins, cyclic olefins, and diolefins have bromine numbers between 63–235, 58–235, 134–237, and 185–352, respectively [28]. Based on these, it can be stated that Py oil-1 is likely to be composed of straight and branched-chain olefins, while the majority of Py oil-2 contains diolefins. Note that bromine numbers for light and heavy naphtha are significantly smaller than pyrolysis oils, indicating the major compositional differences in terms of olefinic content.

Py oil-1 and Py oil-2 were distilled up to 210 °C to obtain naphtha-like fractionations so that their exact compositions can be obtained using PIONA. Fractionated pyrolysis oil samples are more suitable samples for PIONA analysis than their unfractionated counterparts because PIONA analysis is limited to hydrocarbons that have boiling points lower than 210 °C and a carbon number around C_11_ [29]. The distillates were labelled as Py oil-1-F210 and Py oil-2-F210. Bromine numbers of the fraction of pyrolysis oils up to 210 °C are given in Table 3. The distillation did not change the bromine number of Py oil-1, whereas it decreased that of Py oil-2 from 304 to 216, indicating high olefinic content. It should be noted that these numbers are still much higher than the bromine numbers of the naphtha samples.

The PIONA analysis results are given in Table 4. Saturated components (naphthenes, and paraffins) of light naphtha and heavy naphtha are approximately 98% and 91%, respectively, with remaining minor components being olefins and aromatics. The ratio of the saturated components in Py oil-1-F210 and Py-oil-2-F210 are approximately 36% and 35%, respectively, significantly lower than that of the naphtha blends. The majority of components in fractionated pyrolysis oils were found to be cyclic olefins (~44%). A high concentration of olefinic substances is typically obtained when the pyrolysis oil is obtained from PP, as reported in an earlier study by Kusenberg et al. [30]. A high fraction of i-paraffins in pyrolysis oils is attributed to using PP as a feedstock for pyrolysis. If PE without significant branching was used as a feedstock, a higher fraction of n-paraffins would be anticipated since PE is likely to decompose into linear hydrocarbons. Aromatics in pyrolysis oils formed during pyrolysis and their formation cannot be linked to other polymer resins such as polystyrene in the feedstock since virgin PP is used for pyrolysis to produce oils [24,31,32,33,34,35].

When pyrolysis is applied to waste PP, it produced a complex mixture of hydrocarbons with low selectivity to desired products (saturated hydrocarbons) and high selectivity to highly reactive olefins that may form deposits on the cracker walls and aromatics that are precursors for coke formation [35]. Kopinke et al. studied the relative rate of coke formation from a variety of ^14^C-labeled hydrocarbons using tracer experiments under the experimental conditions of steam cracking of naphtha at 810 °C [32,33]. They found that there is a strong correlation between the structure of hydrocarbons and tendency for coke formation. Coking potential of hydrocarbons were found to decrease with increasing degree of saturation. In other words, coking tendency of hydrocarbons increase in the order of alkanes < olefins < acetylenes. In addition, coking tendency of naphthenes, an important component of naphtha, was found to be similar to or slightly higher than paraffins, and lower than olefins. Polycycling aromatic hydrocarbons such as acenaphthylene, 9-methylanthracene, and chrysene were found to have very high coking tendency than monocyclic aromatic compounds such as benzene and toluene. Fractionated pyrolysis oil mixtures composition of which are shown in Table 4 cannot be fed to steam crackers without decreasing olefin levels and aromatic levels to acceptable levels [32,33]. Kopinke et al. recommended acceptable levels of olefins in naphtha to be lower than 5% [32]. However, real light naphtha and heavy naphtha samples taken from the feedstock of ethylene plant and aromatics plants, respectively, of PETKIM contains low amounts (0.1%-0.3%) of olefins. A higher fraction of aromatics can be tolerated in the heavy naphtha since it will be used for aromatics production. For ethylene production, however, aromatic levels should be decreased to prevent coke formation in the steam crackers. The evaluation of pyrolysis oil compositions in terms of tendency for coke formation shows that significant operational issues would arise if these fractionated pyrolysis oils were to be fed to the steam crackers directly without any upgrading. Thus, as a stand-alone technology, pyrolysis oil can neither replace nor be blended with naphtha and is not a viable option for closing the circularity of waste plastics.

## 4. Conclusions

This study focuses on comparing two different pyrolysis oils to light and heavy naphtha, and critically evaluates the suitability of using pyrolysis oil as a feedstock for producing polyolefins to close the gap in the circularity of plastic waste. Liquid samples obtained from the noncatalytic pyrolysis of PP were analyzed by several standardized tests. Analyses were also performed for two different naphtha samples for comparison purposes. The results show that although some of the physical properties such as density, initial boiling point, and sulfur content of the pyrolysis oils and naphtha are similar, pyrolysis oils examined in this study exhibit two major differences than the naphtha samples: (i) pyrolysis oils have a wider carbon distribution than naphtha. This difference, however, can be alleviated by integrating a condenser into the reactor or distilling the pyrolysis oil to obtain the desired carbon range. (ii) Cyclic olefins make up the majority of the pyrolysis oils, whereas the majority of naphtha samples are paraffinic hydrocarbons. If the studied pyrolysis oils are used as feedstock for steam crackers, excessive carbon formation may occur inside the naphtha cracker and operational issues may arise because of reactive unsaturated hydrocarbons present in the pyrolysis oil.

The results demonstrate that the compositional differences prevent pyrolysis oil being used as a substitute for naphtha and a feedstock for steam crackers. Even after distillation, less than 10% of the pyrolysis oil in our study exhibited naphtha-like properties. This, however, does not mean that all pyrolysis oils fall under this conclusion. Pyrolysis oil properties are heavily dependent on system parameters, operating conditions, and catalyst attributes. It is also possible to combine pyrolysis with other chemical technologies to upgrade the properties of pyrolysis oil. Therefore, the findings of our study should not be generalized to mean that pyrolysis oil could never be used as a feedstock for steam crackers to close the gap in the circular economy. However, if pyrolysis oil is used as a feedstock for existing steam crackers and refinery infrastructure, it should exhibit naphtha-like properties. For making such an assessment, this study can serve as a benchmark for evaluating the naphtha-like feedstock properties of steam crackers.

## Figures and Tables

**Table 1:** Density, vacuum distillation, and total sulfur results.

Test	Unit	Light Naphtha	Heavy Naphtha	Py Oil-1	Py Oil-2	Method
Density, 15 °C	g/cm^3^	Min. 0.67, max. 0.72	≥0.73	0.79	0.78	ASTM D4052
Total Sulfur	wt.%	Max. 0.060	Max. 0.10	0.3	0.006	ASTM D5453
IBP *	°C	≥33	≥50	60	55	ASTM D1160
5% Fraction	°C	-	-	97	97	
10% Fraction	°C	-	Min. 85	115	115	
20% Fraction	°C	-	-	145	140	
30% Fraction	°C	-	Min. 105	155	165	
40% Fraction	°C	-	-	195	210	
50% Fraction	°C	Min. 115	Min. 120	232	242	
60% Fraction	°C	-	-	272	280	
70% Fraction	°C	-	Min. 135	325	338	
80% Fraction	°C	-	-	365	372	
90% Fraction	°C	Min. 170	Min. 170	380	385	

* Initial boiling point.

**Table 2:** SIMDIST analysis results.

Mass (%)	Boiling Point (°C)			
	Light Naphtha	Heavy Naphta	Py Oil-1	Py Oil-2
5	36.4	96.0	61.7	66.4
10	40.8	101.5	110.1	123.5
15	-	107.6	132.5	133.2
20	46.0	113.1	133.9	134.3
30	50.7	117.9	136.0	158.9
40	55.6	125.0	190.2	227.6
50	60.6	128.7	234.5	246.9
60	66.6	134.7	275.2	303.4
70	72.2	140.5	320.0	339.7
80	79.1	146.5	374.3	382.4
85	-	150.3	407.2	405.4
90	89.9	151.8	436.2	430.4
95	100.2	157.9	474.0	464.0
96	-	159.1	485.2	472.2
97	-	160.7	495.8	483.6
98	-	163.2	508.9	497.2
99	-	167.1	527.5	517.7
FBP *	117.9	171.4	541.6	537.9

* Final boiling point.

**Table 3:** Bromine number of the pyrolysis oils and naphtha samples.

Sample	Bromine Number (g/100 g)
Light Naphtha	1.2
Heavy Naphtha	0.3
Py oil-1	85
Py oil-2	304
Py oil-1-F210	88
Py oil-2-F210	216

**Table 4:** PIONA analysis summary.

Component	Light Naphtha, % (*w*/*w*)	Heavy Naphtha, % (*w*/*w*)	Py Oil-1-F210, % (*w*/*w*)	Py Oil-2-F210, % (*w*/*w*)
Naphthenes	15.7	36.3	9.3	10.1
i-Paraffins	41.7	28.8	19.1	19.1
n-Paraffins	40.3	25.9	7.8	5.5
Cyclic Olefins	-	-	45.3	43.4
Olefins	0.1	0.3	15.0	16.8
Aromatics	2.2	8.8	3.5	5.2

## Data Availability

Data is contained within the article.
